# An Anthropocentric View of the Virosphere-Host Relationship

**DOI:** 10.3389/fmicb.2017.01673

**Published:** 2017-08-30

**Authors:** Rodrigo A. L. Rodrigues, Ana C. dos S. P. Andrade, Paulo V. de M. Boratto, Giliane de S. Trindade, Erna G. Kroon, Jônatas S. Abrahão

**Affiliations:** Laboratório de Vírus, Department of Microbiology, Universidade Federal de Minas Gerais Belo Horizonte, Brazil

**Keywords:** virosphere, anthropocentric, virus–host relationship, network, metavirome

## Abstract

For over a century, viruses have been known as the most abundant and diverse group of organisms on Earth, forming a virosphere. Based on extensive meta-analyses, we present, for the first time, a wide and complete overview of virus–host network, covering all known viral species. Our data indicate that most of known viral species, regardless of their genomic category, have an intriguingly narrow host range, infecting only 1 or 2 host species. Our data also show that the known virosphere has expanded based on viruses of human interest, related to economical, medical or biotechnological activities. In addition, we provide an overview of the distribution of viruses on different environments on Earth, based on meta-analyses of available metaviromic data, showing the contrasting ubiquity of head-tailed phages against the specificity of some viral groups in certain environments. Finally, we uncovered all human viral species, exploring their diversity and the most affected organic systems. The virus–host network presented here shows an anthropocentric view of the virology. It is therefore clear that a huge effort and change in perspective is necessary to see more than the tip of the iceberg when it comes to virology.

## Introduction

The virology, as a science field, started at the end of the XIX century with the studies of Adolf Mayer, Dmitry Ivanofsky, and Martinus Beijerinck about tobacco mosaic disease. The investigators noticed that they were dealing with an agent completely unknown to the academic community, which retained its infectious nature even after passing through Chamberland filters (at that time, the most efficient method to retain bacteria). Furthermore, even after being diluted by filtration in a porous membrane, the agent recovered its infectiveness after replication within living tissues of healthy plants. The new pathogen was named “*contagium vivum fluidum*,” and only after the advent of *in vitro* plaque assays and electron microscopy it was fully recognized as a virus (Enquist and Racaniello, 2013). Lwoff (1957) published a seminal work in which he established, for the first time, a set of characteristics for an organism to be considered a virus; among them were being an intracellular parasite and completely relying on the biosynthetic machinery of its host, thus being considered a non-living organism. With the advancement of virology, the International Committee on Taxonomy of Viruses (ICTV) was created in the 1960s (originally the International Committee on Nomenclature of Viruses) with the objective of cataloging and organizing the viruses that were being described in the years to come; it established the first rules for viral taxonomy. A few years later, David Baltimore proposed a strategy to organize the viruses according to the properties of their genetic material, with six groups being defined at that time: I (dsDNA), II (ssDNA), III (dsRNA), IV [ssRNA(+)], V [(ssRNA(-)], and VI (ssRNA-RT) (Baltimore, 1971). In the following years, two additional groups were considered, composing the groups VII (dsDNA-RT) and VIII (viroids). This organization strategy is currently well accepted among virologists.

In the years to come, several viruses were described, being isolated in every corner of the planet from hosts belonging to the three domains of life, i.e., Eukarya, Bacteria, and Archaea. In this context, the virus species concept was created by the ICTV, which is the lowest taxon (group) in a branching hierarchy of viral taxa, defined as a polythetic class of viruses that constitute a replicate lineage and occupy a particular ecological niche (i.e., possess similar biological features) (International Committee on Taxonomy of Viruses - Taxonomy, 2017). These viruses continuously reaffirmed the established criteria raised in the 1950s to recognize an organism as a virus. Only during the last few years this paradigm was broken with the discovery of giant viruses (La Scola et al., 2003; Boyer et al., 2009; Philippe et al., 2013; Legendre et al., 2014). These viruses put the well-established concepts to the test, restoring debates about their complete dependency on their hosts and whether they should be considered living organisms, therefore deserving a place in the metaphorical tree of life (Raoult and Forterre, 2008; Forterre, 2010). Besides, advancements in the field of genomics during the last few years, especially metagenomics (or even metaviromics), have allowed the identification of countless viral sequences in several regions of the globe, supporting previous electron microscopy data which suggested the viral ubiquity and an astronomical number of viruses on Earth, thus forming a virosphere (Suttle, 2005; Kristensen et al., 2010).

Although the identification of new viruses and studies of their interaction with hosts have considerably advanced, we still do not know how this interactive network is truly connected. Moreover, many metaviromic studies have been developed allowing the identification of different viral sequences around the world, but we do not have a clear vision of how the viral diversity is distributed on the planet, or how much we have searched for new viruses. Therefore, a new look into what is currently available and the use of new strategies to explore these data could bring new insights and allow the advancement of the virology field. Through extensive meta-analysis of currently available data, we demonstrate here that the known viruses have a very narrow host range, resulting in a spatially connected network. We found a highly anthropocentric view of the virosphere and demonstrated the existence of some specific viral groups in certain environments on the Earth, leading us to reflect about how far we have progressed in the study of viruses. Finally, we analyzed the diversity of human-associated viruses and the tropism of these viruses. The results presented here show a highly biased virology, confirming that we know only the tip of the iceberg and a lot of work remains to be done so we can have a clearer view of the diversity and ecology of the virosphere.

## Materials and Methods

### Dataset Preparation and Selection Criteria

#### Virosphere and Hosts

To analyze the host range of the known viruses, only those officially recognized by the International Committee on Taxonomy of Viruses (ICTV) were included in the analysis. The definition of the best dataset to perform this analysis comprises a challenging task. In this context, ICTV proved to be the best option for gathering the largest and most updated dataset of recognized virus species, grouping and reflecting the diversity and circulation of viruses in nature. A list containing all of the virus species was downloaded from ICTV website^1^. A list released on May 26th, 2016 was used. Therefore, new viruses classified by means of metagenomic data, following the new criteria recently approved by the Executive Committee of ICTV (Simmonds et al., 2017), as wells as the reclassification of the family *Bunyaviridae*, were not considered in this analysis. We considered hosts those organisms in which we found consistent and recurrent evidences of the detection of a virus in a given species by means of isolation, serology, and molecular detection. This detection was associated in most cases with clinical manifestation and, in a few cases, in a non-disease context. Organisms used as study models were not considered here. Hosts were associated with each virus at the lowest taxonomic level possible using the Virus–Host Database (Mihara et al., 2016), VIDE database^2^, and full research articles related to a given virus. In the latter, only one reference was used to determine the host species, even though more than one study (whenever available) was analyzed to corroborate the reference used. During our research and analyses, we considered (whenever the data were available) different viruses within a virus species and their host-range. Only the viruses in which it was possible to determine the hosts at species or genus taxonomic level were considered for the construction of the network. A total of 4497 nodes were included in the network dataset, classified as virus, animalia, plantae, fungi, protist, bacteria, and archaea, along with 4814 edges directly connecting the nodes, all with weight (*w*) = [1].

#### Viral Diversity

To analyze the known viral diversity on the planet, we considered viral groups (families recognized by the ICTV or groups currently unassigned to a proper taxa) identified in diverse metavirome studies performed in the following environments: marine [10], freshwater [7], soil [6], hypersaline [5], thermal springs [4], sewage [4], and polar water [3], in a total of 39 works. The studies were accessed at National Center for Biotechnology Information (NCBI)^3^ using the name of the environments added by virome or metavirome as keywords in the search field. All of the viral groups identified were included in the network analysis, where they were associated with the environments in which they were detected. A total of 103 nodes were included in the network graph, classified according to the analyzed environments and viral order recognized by the ICTV [*Ligamenvirales, Tymovirales, Herpesvirales, Caudovirales, Picornavirales, Mononegavirales, Nidovirales*, and those not classified in order (Unassigned)], and 260 edges indirectly connecting the nodes, with *w* = [1]. To better visualize the viral groups shared between different environments, we created a circular layout image using Circos package (Krzywinski et al., 2009). In addition to the detected viral groups, we computed the type of technology used for nucleic acid sequencing, the type of material analyzed (DNA or RNA), and whether a 200 nm filter was used for sample preparation.

#### Human Viruses and Viral Tropism

The viruses that affect humans were defined after the association of the hosts of each virus species recognized by the ICTV, as described above. The viruses were associated with the following organic systems, according to the clinical manifestation reported in cases of infection: digestive, integumentary, respiratory, nervous, muscular, skeletal, cardiovascular, urinary, reproductive, lymphatic, immune, endocrine, or none of them, in cases of non-pathogenic viruses, based on clinical manifestation and/or tropism for a particular body tissue. Clinical manifestation and the tropism for each system were defined according to full research articles found at NCBI and using the arboviruses catalog of the Center for Disease Control and Prevention^4^. The viruses were associated with different systems in a bipartite network composed of 333 nodes classified according to the organic systems and viruses, and 497 edges indirectly connecting the nodes, with *w* = [1]. In parallel, we built a unipartite network graph wherein the systems were interconnected according to the viruses that affect different systems simultaneously, in a total of 12 nodes and 42 edges indirectly connecting the nodes, with *w* = [1,25].

### Construction of Networks

The networks presented in this work were built using the program Gephi version 0.9.1 (Bastian et al., 2009). All components of the each graph were listed in a comma-separated values (.csv) spreadsheet, which was imported to the software. Another .csv spreadsheet containing the connections between the components was also imported to generate the raw graph. In all networks, the node diameter is directly proportional to the edge degree. The thickness of the edges is directly proportional to the number of times that a node is connected to another, wherein different weights were assigned to the edges. The layout was generated using algorithms based on force of attraction and repulsion of the nodes (Fruchterman-Reingold followed by ForceAtlas 2), followed by local rearrangement of the nodes for a better visualization of the connections between nodes, without perturbing the general layout of the networks.

## Results and Discussion

### The Known Viruses Have a Very Narrow Host Range

**FIGURE 1:**
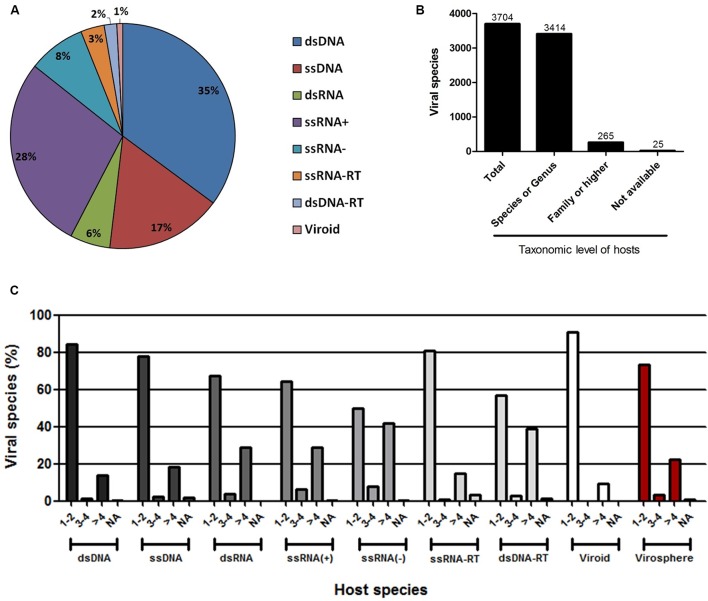
Host range of the known virosphere. **(A)** Pie chart showing the distribution of the viruses recognized by the International Committee on Taxonomy of Viruses (ICTV) according to Baltimore’s classification. **(B)** Taxonomic level of the hosts associated to the known viruses. More than 90% of the viruses were associated to hosts at species or genus taxonomic level, which were used in following analysis. **(C)** Amount of host species for viruses according to Baltimore’s classification, showing a very narrow host range of the viruses. NA, not available.

The ICTV is the organization responsible for cataloging and classifying viruses into virus species that have been described over time. Historically, this organization has taken into consideration several criteria for a new isolate to be considered a new species, such as the genetic material and the hosts in which it was isolated, as well as any clinical manifestations it may possibly cause (Simmonds et al., 2017). Viral taxonomy covers the levels of order, family (and subfamily in some cases), genus and species, wherein the vast majority of virus species remain outside of a virus order. All of this information is constantly updated by the ICTV, which periodically publishes the Master Species List (MSL). In this work, we evaluated the host range of all known viruses with a virus species officially recognized and published by the ICTV on May 26th, 2016 (MSL#30) [**Supplementary Table S1**]. An extensive search using public databases and indexed publications was performed to define the natural hosts of all of the viruses present in the list (see Materials and Methods). The majority of the viruses present in the MSL#30 (a total of 3704 virus species, henceforward named the known virosphere) comprises group I (dsDNA) and IV [ssRNA(+)] according to Baltimore’s classification [35 and 28%, respectively, followed by group II (ssDNA – 17%)], with the remaining groups representing 20% of the known virosphere (**Figure 1A**). It was possible to associate hosts at the species or genus level to 3414 viruses (92.2%), at the family level or higher to 265 viruses (7.15%), and it was not possible to associate any host for only 25 viruses (0.65%), either because the natural hosts for the viruses are not yet known, or due to a complete lack of information in the literature about their host range (**Figure 1B**). For all viral groups, according to Baltimore’s classification, the host range is very restricted, with more than 50% of known viruses infecting only one or two host species, reaching up to 75% in some groups, such as those viruses with genomes composed of dsDNA, ssDNA, ssRNA-RT, and viroids (**Figure 1C**). Only the ssRNA(-) viruses seems to possess a slightly broader host range, wherein 42% of the viruses are able to infect more than four host species. Considering the entire known virosphere, 73.3% are associated with only one or two host species; 3.5% with three or four species; 22.5% with more than four species; and only 0.7% have a natural host range which has not been defined (**Figure 1C**). These analyses reveal that, until now, based on the available information we have, viruses have a very narrow host range. This disturbing data must be interpreted carefully. It is likely that several unknown viruses have a broader host-range, which will drastically change the view presented here; however, we might be far from acquire this kind of knowledge since these relationships are likely out of scope of human investigation. Therefore, in light of the research performed so far, we are facing such suspicious data.

### An Anthropocentric View of the Known Virosphere

**FIGURE 2:**
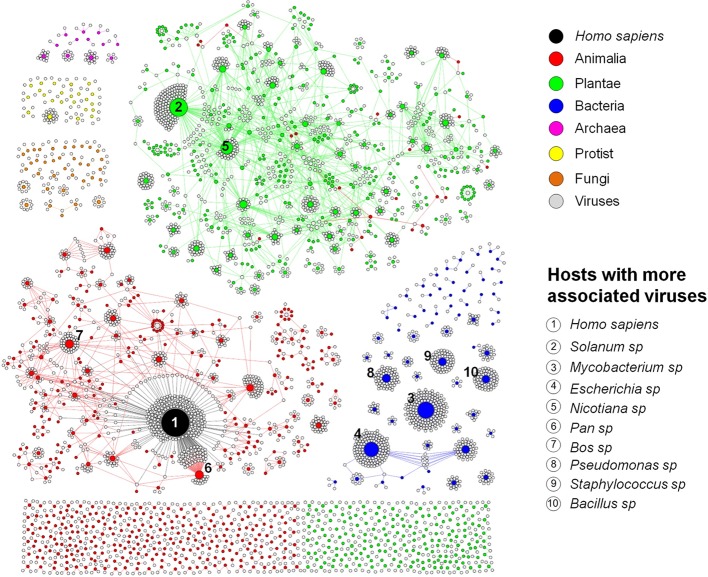
Virus–host interaction network. Bipartite network graph showing a spatially connected network among viruses and hosts, a reflection of our limited knowledge about the viruses and their hosts. Each node represents a virus (gray), or a host genus, classified according to the taxonomic group (colored nodes). The nodes’ diameter is proportional to the edge degree. The layout was generated using a force based algorithm followed by manual rearrangement to a better visualization of the connections. A total of 3414 viruses and 1083 hosts (genus level) are represented. The 10 hosts with more associated viruses are depicted.

To better represent the interaction between the viruses and the hosts so that we can have a clear vision of how interconnected these organisms are, we built a bipartite network graph composed of 4497 nodes, with 3414 viruses (only viruses associated with hosts at species or genus taxonomic level were included in this analysis) and 1083 hosts (at genus level), all connected by 4814 edges with the same weight (*w*) = [1]. The hosts were classified according to the major realms and domains of life: Animalia, Plantae, Protist, Fungi, Bacteria, and Archaea (Woese, 2002). We observed a spatially connected network, wherein only a few hosts were associated to a huge amount of viruses, while the majority of the hosts are associated with a few viruses, a reflex of the very narrow host range of the known virosphere (**Figure 2**). Furthermore, the analysis of the network revealed a highly anthropocentric virosphere, in which most viruses are associated with humans or hosts that are directly related to humans by economic, medicinal or biotechnological interests. The vast majority of known viruses are associated with plants (483 genera) or animals (467 genera). These groups are more interconnected than others, even though more than 70% of these hosts possess only one or two associated viruses (**Supplementary Figure S1**). It is noteworthy that some viruses can cross broad host categories, infecting both plants and animals. These viruses are plant pathogens transmitted by arthropod vectors, in which are able to fully replicate and reach the plant host (Dietzgen et al., 2016). Bacteria-infecting viruses (known as bacteriophages or phages) are mainly distributed among the families *Myoviridae*, *Podoviridae*, and *Siphoviridae* (order *Caudovirales*), and are associated with 62 known host genera. This group is spatially connected, reflecting the narrow host range of phages. However, different to animals and plants, almost 40% of known bacteria are infected by more than four viruses. Some bacteria comprised hubs in the network, such as *Mycobacterium* and *Escherichia*, with several associated viruses. Since they are intensively studied due to their medicinal and biotechnological relevance (Korb et al., 2016; Vila et al., 2016), it was expected that a large number of viruses would be identified as parasites of these groups. In fact, a large majority of phage sequences available in GenBank was isolated from a few groups of bacteria associated to human diseases or food processing (Holmfeldt et al., 2013). The knowledge about viruses affecting fungi, protists and archaea is scarce, probably due to the lack of investigation of these groups of viruses and their hosts. These viruses were associated with 36 genera of fungi, 23 protists, and only 12 genera of archaea, reflecting how poorly these microorganisms are studied under the lens of virology.

Among the host genera of each group that possess more associated viruses, many are composed of domesticated species such as *Bos* sp., *Sus* sp., and *Gallus* sp. (Animalia; e.g., cattle, swine, and chickens, respectively); *Solanum* sp., *Nicotiana* sp., *Phaseolus* sp., *Capsicum* sp., and *Cucumis* sp. (Plantae; e.g., potato, tobacco, common bean, peppers, and cucumber, respectively); *Chlorella* sp. (Protist); and *Saccharomyces* sp. (Fungi) (**Supplementary Figure S2**). Many species of these groups are employed in farming, such as cattle, pigs and poultry, as well as many grains and legumes consumed worldwide, handling billions of dollars annually (Thornton, 2010; Reganold and Wachter, 2016). In addition, some species of green algae (*Chlorella* sp., *Chlorophyta* phylum) are used as dietary supplementation as sources of vitamins and macro-nutrients and its efficacy against some human diseases are under constant investigation (Ebrahimi-Mameghani et al., 2016; Panahi et al., 2016). Yeasts of the *Saccharomyces* genus, especially *S. cerevisiae*, are considered domesticated fungi, being used worldwide in the production of alcoholic beverages, also making them economically important (Sicard and Legras, 2011; Gallone et al., 2016). Given the economic relevance of these organisms, constant efforts are made to reveal parasites that might be considered a threat to them, thus enabling possible strategies of control and prevention to be established. Therefore, it was expected that these groups of hosts had more known viruses.

Other hosts are known due to their medicinal relevance for humans or animals and commercially explored plants, such as *Acanthamoeba* sp. and *Trichomonas* sp. (Protist), both related to severe infections in humans (Siddiqui and Khan, 2012; Menezes et al., 2016); *Heterobasidion* sp., *Cryphonectria* sp., *Rosellinia* sp., and *Ophiostoma* sp. (Fungi), groups of fungi related to diverse plant infections, both domesticated and from native forests, causing severe diseases such as annosum root and chestnut blight (Hillman and Suzuki, 2004; Ďurkovič et al., 2013; Kondo et al., 2013; Vainio and Hantula, 2015); and *Mycobacterium* sp., *Escherichia* sp., *Pseudomonas* sp., *Staphylococcus* sp., and *Bacillus* sp. (Bacteria), all groups of prokaryotes related to life-threatening diseases, such as tuberculosis (Korb et al., 2016), gastrointestinal, respiratory and urinary infections (Langan et al., 2015; Vila et al., 2016), and also used as biological weapons (Goel, 2015). Therefore, it is expected that these species are the target of intense investigation, and the majority of known phages are associated with these bacteria. Finally, some hosts are important in the biotechnology field or used as laboratory study models for molecular biology, such as *Ectocarpus* sp. (Protist) (Lipinska et al., 2016); *Sulfolobus* sp., and *Thermus* sp. (Archaea) (Cava et al., 2009; Zhang et al., 2013) (**Supplementary Figure S2**). Altogether, the data presented here show that in all group of hosts, both eukaryotic and prokaryotic, most of the known viruses are related to hosts that are important for humans in certain aspects. In this way, the virus–host network shows a highly anthropocentric view of the virology performed so far. This biased virology is probably the very reason for our view of a narrow host-range of the known viruses.

### Viral Diversity on Earth

Since the discovery of the tobacco mosaic virus at the end of XIX century, many other viruses have been described and biologically characterized in many regions of the planet, thus contributing to the concept of viral ubiquity. With advances in electron microscopy techniques, many studies have been conducted in order to define the abundance and diversity of viruses, coming to an astronomic number, in the order of 10^31^ viral particles on the Earth (Suttle, 2005). However, only with the advent of massive parallel sequencing of nucleic acids and the development of a new research field – metagenomics – it was possible to create a better view of the viral diversity on the planet, reaffirming the viral ubiquity concept (Kristensen et al., 2010).

**FIGURE 3:**
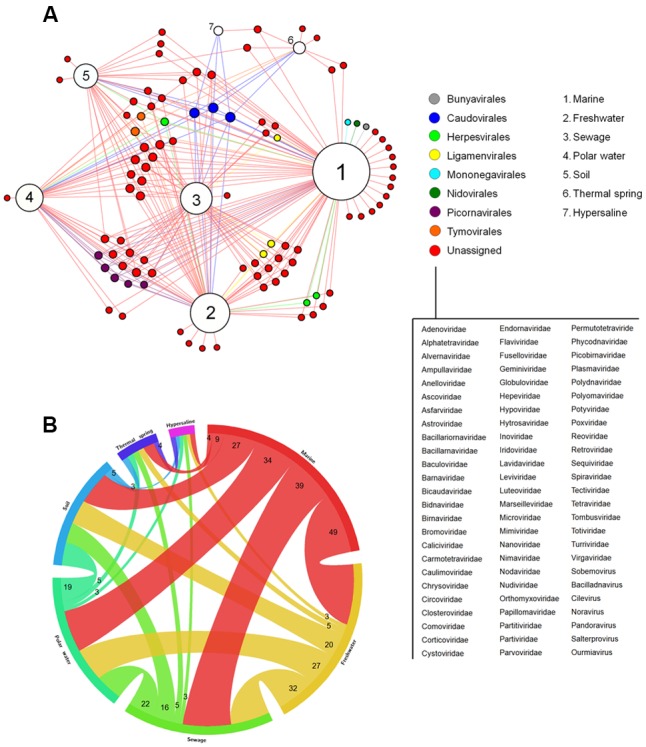
Viral diversity on Earth. **(A)** Network graph showing the viral groups detected in different environments by metaviromic analysis. Each node represents an environment (white) or viral groups (families or known viral genus – colored nodes) classified according to the orders formally recognized by the ICTV. The viruses not currently assigned in any order are listed. The node diameter is proportional to the edge degree. The layout was generated using a force based algorithm followed by manual rearrangement for a better visualization of the connections. A total of 96 viral groups are represented. **(B)** Relationship between the different environments based on the amount of shared viral groups.

By analyzing different available metagenomic works, more specifically metaviromic works (analysis of viral nuclei acid sequences in different environments), we built a bipartite network graph connecting the viral groups found within seven distinct environments around the planet: marine, freshwater, polar water, thermal springs, hypersalines, and sewage (**Figure 3A**). A total of 39 works were analyzed (for choice criteria, see Materials and Methods). A total of 96 viral groups (genus or family) were detected in those studies. Different amount of viral groups are shared among the environments, wherein marine shared up to 49 viral groups with other environments, reinforcing the ubiquity of viruses on the planet (**Figure 3B**). Among the viral groups identified, only representatives of the families *Myoviridae*, *Podoviridae*, and *Siphoviridae* (phages belonging to the order *Caudovirales*) were found in all of the searched environments. After the initial studies of metagenomics in marine environments, in which they searched basically for bacteriophages, the hypothesis “Everything is everywhere but environment selects” was applied to these viruses, stating the ubiquity of the phages, even though some groups were specifically found in certain environments (O’Malley, 2008; Thurber, 2009). Our meta-analysis corroborates this hypothesis and goes further, showing that head-tailed phages are found in every location investigated, not only in marine samples. In contrast, the majority of viral groups were found only in two or three environments, and surprisingly, some groups were also restricted to only one environment (**Figure 3A**). The viral diversity is higher in marine environments, wherein 15 groups were exclusive to it. The great diversity of viruses in the oceans is a reflection of the abundance of hosts found there, but also reflects the number of studies performed, covering all of the oceans and many important seas around the globe, such as the Mediterranean, the Baltic and the Arctic (**Supplementary Table S2**). As expected, extreme environments, such as thermal springs (high temperatures) and hypersalines (high osmolarity), were those with the lowest viral diversity, with only 11 and four viral groups found in each, respectively. The families *Globuloviridae* and *Spiraviridae* were detected exclusively in thermal springs. The viruses of these families infect hyperthermophilic archaea, which are highly abundant in hot springs, thus explaining the exclusivity of those viruses in these environments. No viral group was exclusive to hypersaline environments. Curiously, viruses belonging to the families *Sphaerolipoviridae* and *Pleolipoviridae* (archaea-infecting viruses) have already been isolated and characterized from extreme environments (Luk et al., 2014); however, representatives of these groups were not detected by metaviromic approaches so far.

The absence of some viral groups in certain metaviromic studies might be due to the employed methodology, either in the sequencing platform/method and bioinformatic pipelines, in the type of genetic material that was analyzed (DNA or RNA), or even (and mainly) the procedures employed in the preparation of the samples for sequencing. The vast majority of studies target DNA viruses and use 0.2 μm porous filters during the processing of the collected samples (**Supplementary Table S2**). These strategies restrict the detection of a large part of the viruses (those with RNA genome) and also the giant DNA viruses (Halary et al., 2016), thus making a change in the protocols for the preparation of samples for metaviromic approaches necessary. Nevertheless, it is important to emphasize that the majority of the sequences found in metaviromic studies has no similarities with known sequences available from public databanks. This demonstrates that although the emergence of metagenomic techniques greatly contributed to the discovery of new viruses, even leading the ICTV executive committee to recently approve the use of such information for viral classification (Simmonds et al., 2017), the works on isolation and characterization, both genomically and biologically, should continue and be encouraged. With the association of biological/virological and metaviromic approaches, we might have new insights into the real diversity and distribution of viruses on Earth.

### Human-Associated Viruses and Viral Tropism

**FIGURE 4:**
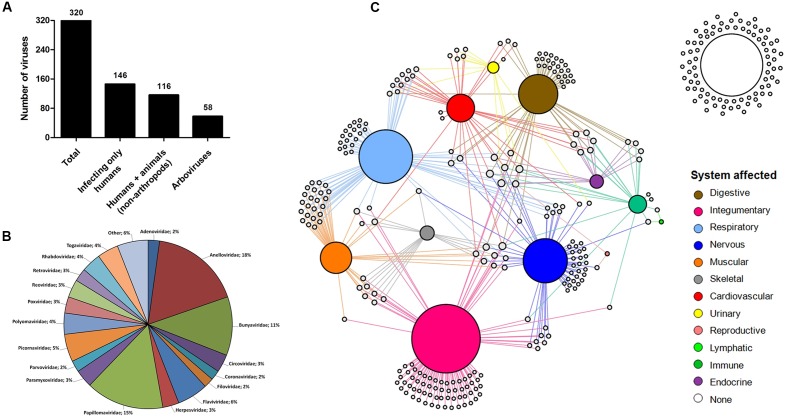
Human viruses and affected systems. **(A)** Human-affecting viruses divided among infecting only humans, infecting humans and other mammals, and arboviruses. **(B)** Pie chart showing the classification of the viruses. A total of 27 groups are represented in the chart. Others: *Deltavirus, Hepadnaviridae, Hepeviridae, Caliciviridae, Picobirnaviridae, Pneumoviridae, Arenaviridae, Orthomyxoviridae, Astroviridae*. **(C)** Network graph showing the viral tropism. Each node represents a virus (white) and an organic system of the human body (colored nodes). The node diameter is proportional to the edge degree. The layout was generated using a force based algorithm followed by manual rearrangement to a better visualization of the connections.

Since human species is the one with more associated viruses officially recognized by the ICTV among all of the hosts analyzed here, the next step was to turn our attention to these viruses. Until recently, it was thought that about 200 viruses were associated with infections in humans, some with no direct evidence of causing any disease (Woolhouse et al., 2012). Here, we demonstrate that among the known virosphere, 320 virus species are related to human infections (**Supplementary Table S3**). Among them, 146 (45.6%) infect only humans; 116 (36.2%) infect humans and other mammals, some considered important zoonosis, such as rabies (*Rabies lyssavirus*), poxviruses (*Orthopoxvirus*), and hantaviruses (*Hantavirus*) (Shchelkunov, 2013; Jackson, 2016b; Jiang et al., 2017); and 58 (18.2%) are arboviruses (viruses transmitted by arthropods, including mosquitoes, sandflies and ticks) (**Figure 4A**). These viruses are classified within 26 families, wherein *Anelloviridae*, *Bunyaviridae*, and *Papillomaviridae* are the most significant, gathering 44% of the human viruses (**Figure 4B**). These viruses are highly variable, both structurally and genetically, using different replicative strategies. Although all groups of Baltimore’s classification possess representatives of human viruses [except for viroids that infect only plants (Steger and Perreault, 2016)], the majority belong to groups I–V, with retroviruses accounting for less than 3% of viruses (**Supplementary Table S3**). Although they are the minority among human viruses, retroviruses were central to the emergence of mammals, thus also to humans, being pivotal components in placenta development (Chuong, 2013). In addition, the human immunodeficiency virus (HIV), the main representative of the group, is one the main life-threatening pathogens, being responsible for immunosuppressive conditions, paving the way to numerous severe secondary infections such as tuberculosis, systemic mycosis, Kaposi sarcoma, among others (Miceli et al., 2011; Godfrey-Faussett and Ayles, 2016; Govindan, 2016).

Many viruses are responsible for severe clinical manifestations, while others are related only to mild symptoms of disease or even asymptomatic infections. To have a better view of the tropism of human viruses and the most affected organic system, we built a network graph associating the viruses with different systems of the human body, according to clinical manifestations related to different viral infections. The viruses that have no direct evidence of causing disease were also included in the analysis. The integumentary, respiratory, and nervous systems were the main affected systems, with 92, 72, and 58 associated viruses, respectively (**Figure 4C**). The integumentary and respiratory systems are the most exposed to infection by different micro-organisms, since they are in direct contact with the environment, thus being expected to be the most affected by viruses. It is noteworthy that many viruses that affect the respiratory tract also affect the muscular system, a reflection of the viruses that cause only flu-like symptoms (**Supplementary Figure S3**). Unlike the two first systems, the nervous system is not directly exposed to the environment, thus making it curious that it is the third most frequently affected system by viruses. Since it is an extremely important and delicate system of the human body, several studies have been conducted to elucidate possible threats for its components, leading to the identification of a considerable range of viruses associated with diseases of the nervous systems. Many of these viruses are associated with severe cases of encephalitis and meningitis, such as herpesviruses (Granerod et al., 2010), lyssaviruses (Jackson, 2016a), and flaviviruses (Daep et al., 2014) (**Supplementary Table S4**), which is why they are target of intense investigation, to better understand the biology of these viruses, thus allowing the development of control mechanisms and possible treatments for diseases. Many of the viruses of the nervous system also affect others, mainly the respiratory and integumentary systems (**Supplementary Figure S3**). In that sense, some viruses are considerable pantropics, affecting different systems simultaneously, such as ebolavirus, dengue virus and rubella virus, affecting the cardiovascular (hemorrhagic fever), muscular (myalgia), skeletal (arthralgia), and nervous (encephalitis) systems, among others (**Supplementary Table S4**).

The reproductive and lymphatic systems are the least affected by viruses. The first is affected by only two viruses (mumps virus and Rio Bravo virus), responsible for cases of orchitis and oophoritis (Volkova et al., 2012). Although the herpesviruses and papillomaviruses are commonly associated with infections in the reproductive system, where they cause ulcerative lesions and warts in genital regions, we associated these viruses to the integumentary system, since their tropic site of infection is epidermal cells and not specific organs belonging to the reproductive tract. The lymphatic system has also only two associated virus species (*Human gammaherpesvirus 4* and *Primate T-lymphotropic virus 1*), both related to lymphoma cases. Although some viruses trigger lymph node inflammation, these are not considered the tropic site of infection for most viruses, so they are excluded from this analysis. It is possible that other viruses are related to these systems, as well as others included in this network, but further investigations are required. More studies are necessary regarding these systems, thus we can identify the viruses with tropism for these sites. Finally, 83 (26%) viruses analyzed in this work are not connected to any system since they are not related to any known disease so far (**Figure 4C**). The majority of these viruses belong to the family *Anelloviridae* (67.5%), which is mainly composed of the torque teno viruses. These viruses are present in most parts of people, as many metaviromic studies have demonstrated, but there is still no consensus that they carry any kind of loss for our health. As far as we know, they are part of the human virome along with many bacteriophages (Rascovan et al., 2016). Along with the anelloviruses, others have already been detected in human beings by metagenomic approaches, where the association with any disease remains under discussion, such as the giant mimiviruses and marseilleviruses (Popgeorgiev et al., 2013). While there is some evidence linking these viruses with human pathologies, we are still far from ending this debate.

## Conclusion

It has been more than a century since the discovery of the first viruses. During this time, we have seen great advances in cellular and molecular biology and genetics, which have boosted achievements in the field of virology. Nevertheless, the results presented here show us that, even with great advances, we still know only a tiny fraction of the viral universe, mainly regarding the virus–host interaction. The discovery of giant viruses during the last decade was essential for us to realize how diverse and intriguing the virosphere is, triggering the search for new viruses in hosts completely ignored in the lens of virology. A break of concepts was established after those discoveries, taking us to think again what a virus is and what else is waiting to be discovered. Moreover, the advent of metaviromics had a unique contribution to the expansion of our knowledge about the virosphere, mainly on the diversity and distribution of these microorganisms, but also with the discovery of new viruses (Alavandi and Poornima, 2012; Shi et al., 2016). However, we are still unable to define the host range of these new viruses with enough accuracy based only on genomic data. In that sense, the improvement of viral isolation techniques is important so that we can look deeper into how these new organisms interact with their hosts and the environment which they inhabit.

The analyses shown here provide a picture of what we know about the entire virosphere and their hosts, and confirm the anthropocentric view of the virology so far. It is likely that the network presented here (**Figure 2**) is largely more interconnected. However, further studies should be performed, especially searching for viruses in hosts that are not of primary human interest, such as environmental fungi and archaea, or even plants and animals that have no added medicinal or economic value. It is an arduous work, but with the improvement of viral isolation techniques and metaviromics, both fundamental tools to this task, it will be possible to continuously add new pieces to fulfill the virus–host network, providing a broader view of the viral universe. In that moment, possibly when science would once again be performed and applied to the understanding of the nature rather than serving the exclusive interests of human beings, we might see beyond just the tip of the iceberg.

## Author Contributions

RR, AA, and PB prepared the dataset. RR performed the analysis. RR wrote the manuscript. GT, EK, and JA designed the study. All authors read and approved the final version of the manuscript.

## Conflict of Interest Statement

The authors declare that the research was conducted in the absence of any commercial or financial relationships that could be construed as a potential conflict of interest.

## Footnotes

1 https://talk.ictvonline.org/files/master-species-lists/

2 http://sdb.im.ac.cn/vide/sppindex.htm

3 https://www.ncbi.nlm.nih.gov/pubmed/

4 https://wwwn.cdc.gov/arbocat/

## Supplementary Material

FIGURE S1 Amount of viruses associated by hosts (at genus level) separated by taxonomic group of the hosts. The total amount of hosts is depicted in the top of each column.

FIGURE S2 The five hosts with more associated viruses for all six major taxonomic groups, evidencing that most of them is related to human interests. **(A)** Animalia, **(B)** Plantae, **(C)** Protist, **(D)** Fungi, **(E)** Bacteria, **(F)** Archaea. d, domesticated host; i, infection related host; b, biotechnology application host.

FIGURE S3 Unipartite network graph showing the connections between organic systems according to the viruses that have tropism for more than one system. The nodes’ diameter is proportional to the edge degree. The layout was generated using a force based algorithm followed by manual rearrangement to a better visualization of the connections. The thickness of the edges is proportional to the number of viruses that affect the two systems it connects.

TABLE S1 Viruses and their hosts.

TABLE S2 Technical information of metaviromic works.

TABLE S3 Human-infecting viruses and other animals.

TABLE S4 Tropism of human-infecting viruses and clinical manifestation.
