# Supplementary figures and images for: An Anthropocentric View of the Virosphere-Host Relationship
Source: Front Microbiol. 2017 Aug 30;8:1673. doi: 10.3389/fmicb.2017.01673 (PMC5582082; doi:10.3389/fmicb.2017.01673)

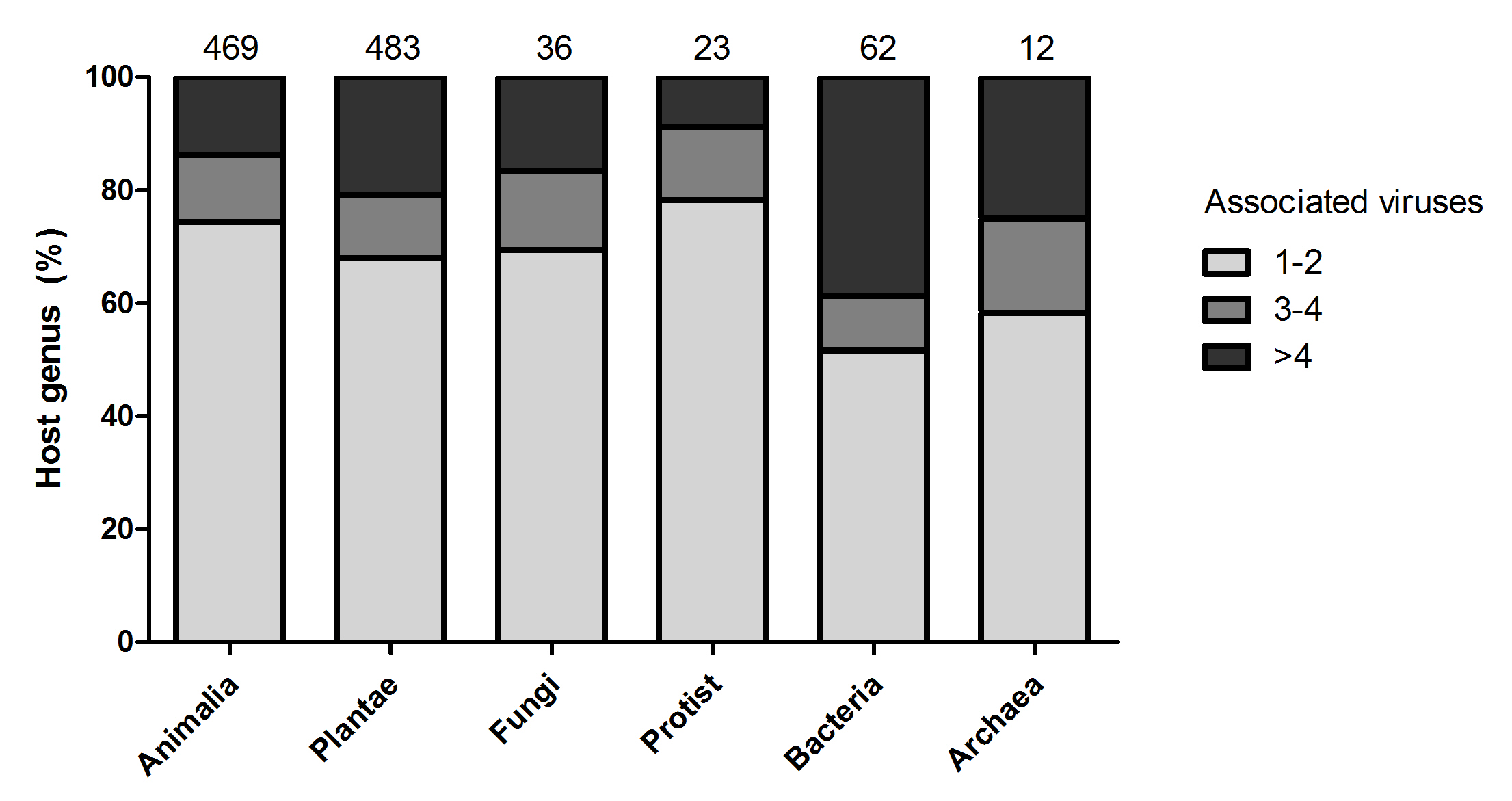

Supplement: FIGURE S1 — Amount of viruses associated by hosts (at genus level) separated by taxonomic group of the hosts. The total amount of hosts is depicted in the top of each column. [file Image_1.TIF]

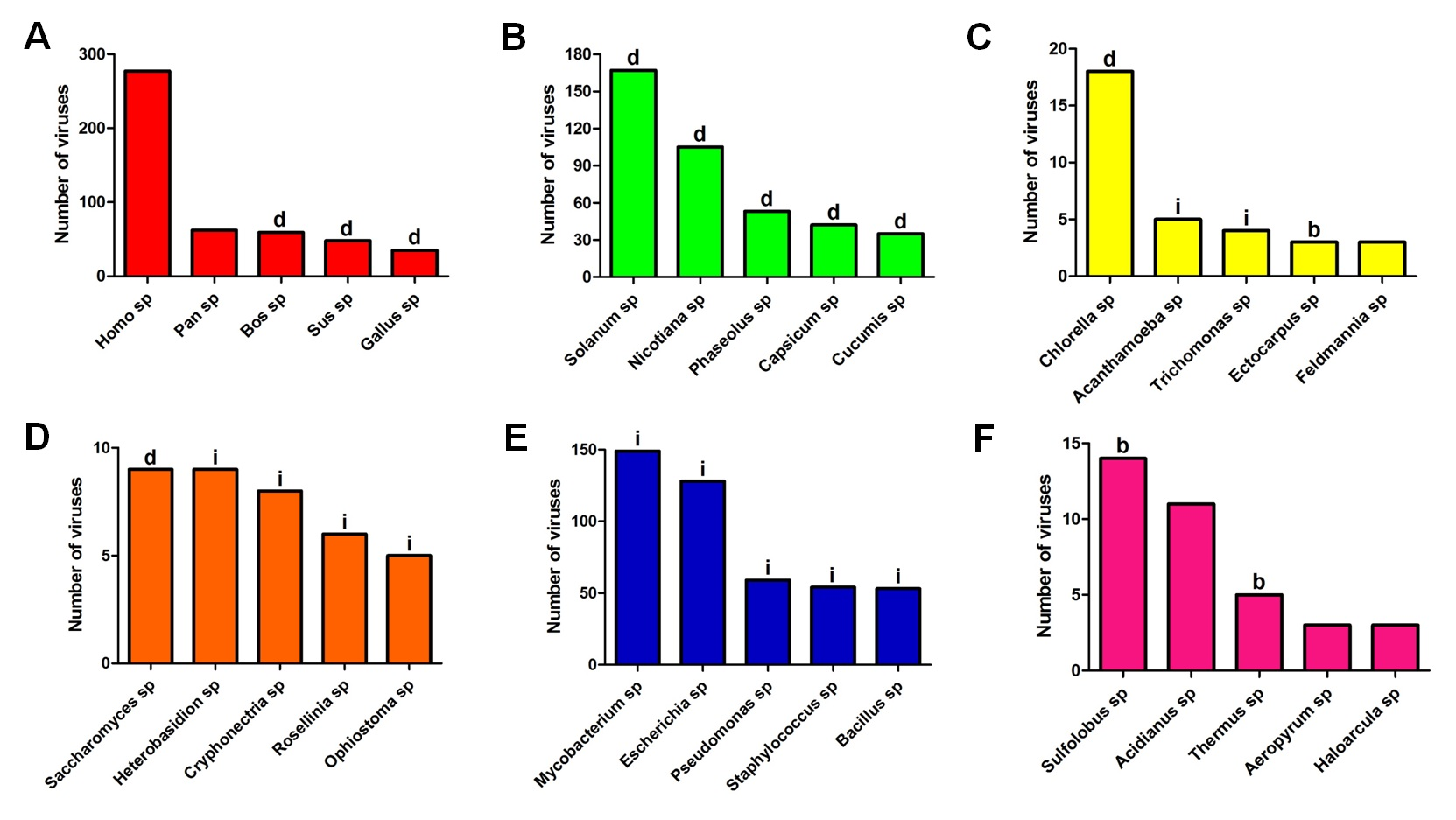

Supplement: FIGURE S2 — The five hosts with more associated viruses for all six major taxonomic groups, evidencing that most of them is related to human interests. (A) Animalia, (B) Plantae, (C) Protist, (D) Fungi, (E) Bacteria, (F) Archaea. d, domesticated host; i, infection related host; b, biotechnology application host. [file Image_2.TIF]

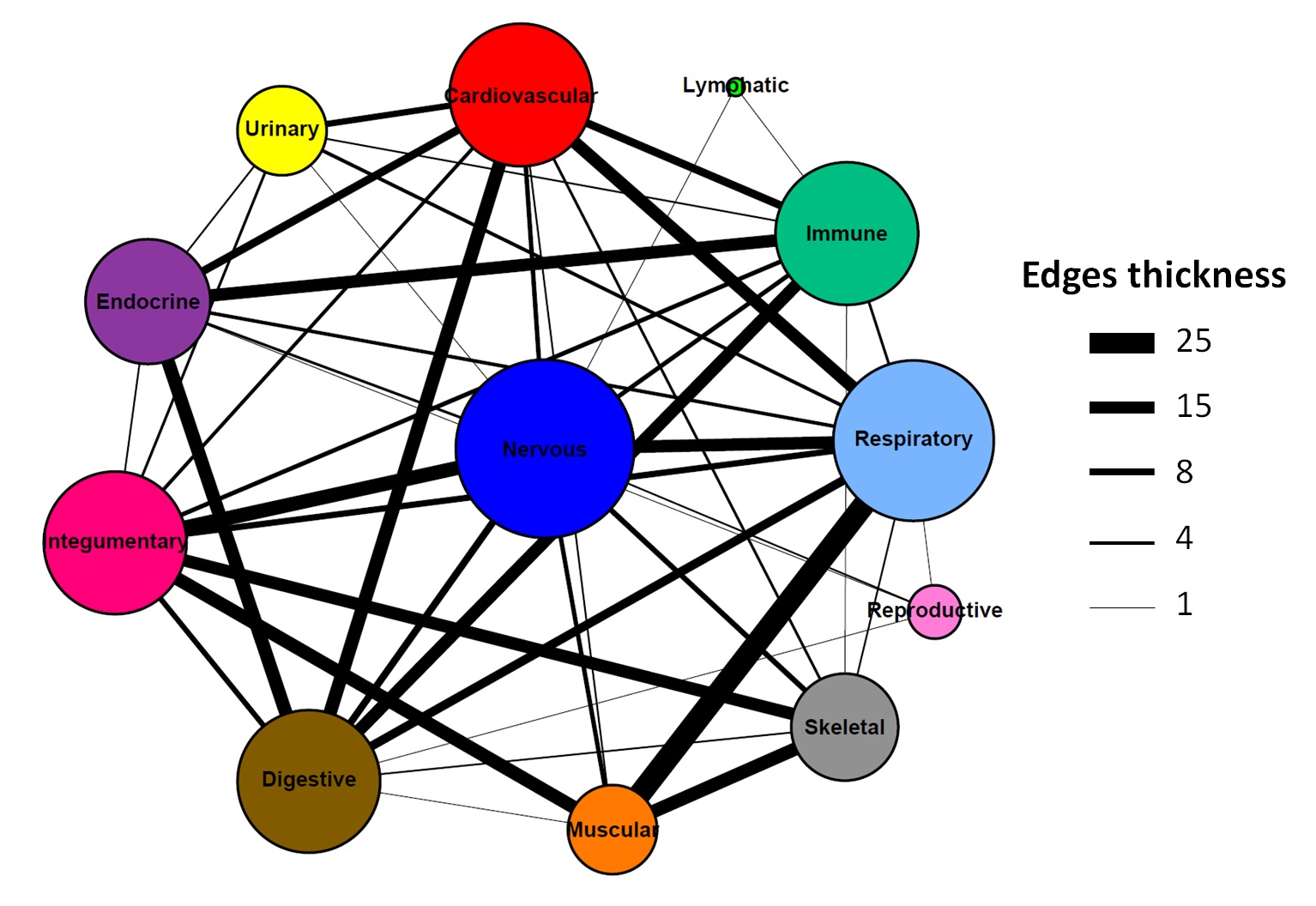

Supplement: FIGURE S3 — Unipartite network graph showing the connections between organic systems according to the viruses that have tropism for more than one system. The nodes’ diameter is proportional to the edge degree. The layout was generated using a force based algorithm followed by manual rearrangement to a better visualization of the connections. The thickness of the edges is proportional to the number of viruses that affect the two systems it connects. [file Image_3.TIF]
